# Women are less likely than men to achieve optimal glycemic control after 1 year of treatment: A multi-level analysis of a Korean primary care cohort

**DOI:** 10.1371/journal.pone.0196719

**Published:** 2018-05-02

**Authors:** Seung-Ah Choe, Joo Yeong Kim, Young Sun Ro, Sung-Il Cho

**Affiliations:** 1 Department of Obstetrics and Gynecology, CHA University, School of Medicine, Gyeonggi-do, Republic of Korea; 2 Department of Epidemiology, Graduate School of Public Health, Brown University, Providence, RI, United States of America; 3 Department of Emergency Medicine, Korea University Ansan Hospital, Gyeonggi-do, Republic of Korea; 4 Laboratory of Emergency Medical Services, Seoul National University Hospital Biomedical Research Institute, Seoul, Republic of Korea; 5 Department of Epidemiology, Graduate School of Public Health and Institute of Health and Environment, Seoul National University, Seoul, Republic of Korea; Universidad Miguel Hernandez de Elche, SPAIN

## Abstract

We investigated differences in the achievement of glycemic control among newly diagnosed type-2 diabetes patients according to gender using a multi-clinic retrospective cohort study. Optimal glycemic control was defined as hemoglobin A1c (HbA1c) of less than 6.5% after 1 year of diabetes management. A generalized linear mixed model, which controlled for the fixed effects of baseline characteristics and prescribed oral hypoglycemic agent (OHA), was used to calculate the probability of achieving the target HbA1c. The study included 2,253 newly diagnosed type-2 diabetes patients who completed 1 year of diabetic management, including OHA, in the 36 participating primary clinics. Within the study population, the women had an older average age, were less likely to smoke or drink alcohol, and showed lower levels of fasting blood glucose and HbA1c at the time of diagnosis. There were no significant differences by sex in prescribed OHA or median number of visits. After 1 year of diabetes management, 38.9% of women and 40.6% of men achieved the target HbA1c—a small but significant difference. This suggests that type-2 diabetes is managed less well in women than in men.

## Introduction

Diabetes mellitus is a chronic endocrine disease that occurs in up to 9% of adults (age of ≥ 18 years) worldwide [1]. In a rapidly aging society, the number of diabetic patients is expected to double by 2030 relative to that in 2000 [2]. Although the age-specific prevalence of diabetes is higher for men than women, there are more women than men with diabetes because women tend to live longer [2].

In total, 90% of diabetic patients are diagnosed with type-2 diabetes mellitus, formerly called non-insulin-dependent diabetes [1]. Type-2 diabetes requires successful glycemic control to prevent microvascular and macrovascular complications [3, 4]. To date, several researchers have suggested that the clinical presentation and prognosis of diabetes differ between women and men [5–10]. At the time of diagnosis of type-2 diabetes, diabetic retinopathy is more frequent and severe in men than in women [10]. On the other hand, women with diabetes show a higher risk of developing coronary heart disease [9]. After adjusting for conventional risk factors, female diabetes patients were found to be more likely than men to suffer from coronary heart disease and subsequent mortality [6–8].

The difference between the sexes in the clinical presentation and prognosis of diabetes may be attributed to sociocultural factors, in addition to biological characteristics [11]. Differences have been reported in the pattern of diabetes care between women and men [12]. Considering the substantial individual variation in long-term compliance with and management of care, a study of treatment outcome following 1 year of diabetes management may provide less biased results. However, there has been scant research on sex differences in the glycemic control of newly diagnosed patients. Hence, we investigated sex differences in glycemic control after 1 year of diabetes management, which included treatment with an oral hypoglycemic agent (OHA), among newly diagnosed type-2 diabetic patients in primary clinics.

## Materials and methods

### Study design and data collection

This investigation was a retrospective cohort study that ran from September 1, 2013 through July 31, 2014. The target population was patients with newly diagnosed type-2 diabetes, drawn from a total of 42 primary clinics across all nine provinces of Korea. Convenience sampling, in which individuals within the target population are selected for the study until the required sample size is reached, was used. Based on a recent study revealing that 1-year adherence to antihyperglycemic medication was negatively associated with risk of hospitalization and premature mortality in diabetes patients [13], the outcome measure was the glucose profile after 1 year of OHA treatment. The physicians were instructed to include newly diagnosed drug-naïve type-2 diabetes patients who completed 1 year of diabetic management, including OHA treatment, at the corresponding primary practice during the previous 3 years (from September 1, 2010 to August 31, 2013). To gather the relevant clinical data, a case report form (CRF) was developed based on potential risk factors of diabetes management from literature review of prior studies. To standardize data collection, all physicians at the participating primary practices received on- and off-line training in CRF administration and the study protocol. After the training and pilot tests, the data recorded into the CRFs by the participating physicians were collected. Demographic data included age at the time of diagnosis (years), sex and Medical Aid use status; Medical Aid is a public health care program for low income people covering 3–4% of the population [14, 15]. Body mass index (BMI; kg/m^2^), diabetes symptoms, medical history at time of diagnosis, fasting blood glucose (mg/dL) and hemoglobin A1c (HbA1c; %) levels and initial prescriptions were retrieved from patients’ medical records. All CRFs that did not adhere to the administration protocol, or were incomplete, were excluded from the final analysis.

Diagnostic criteria for type-2 diabetes followed the Korean Diabetes Association (KDA) guidelines: (1) HbA1c over 6.5%, or (2) fasting blood glucose over 120 mg/dL, or (3) postprandial 2-hour oral glucose tolerance test over 200 mg/dL, or (4) random glucose level over 200 mg/dL and at least one other diabetes symptom (including polydipsia, polyuria, polyphagia, and unexplainable weight loss). Patients who were younger than 18 years, were pregnant, had acute infections that could affect blood glucose control, were unable to use OHAs, had undergone a surgical procedure for malignant tumors within 5 years, or were lost to follow-up were excluded.

All patients included in the study were initially prescribed OHAs. The OHAs were categorized into three types of monotherapy (metformin, sulfonylurea and other monotherapy) and three single-pill combinations (metformin+sulfonylurea, metformin+DPP-IV inhibitor, and other single-pill combinations). At each follow-up visit, HbA1c and fasting blood glucose were measured. The treatment target (i.e., the primary outcome measure) was an HbA1c level below 6.5%, as recommended by the KDA [16, 17].

The study protocol was reviewed and approved by the institutional review board of the Graduate School of Public Health, Seoul National University (IRB No. 54-2013-10-01).

### Statistical analysis

The required sample size was calculated based on the results of our previous report.[18] Assuming that an equal number of female and male diabetic cases would be sampled, 1,830 cases (915 women and 915 men) were needed. This sample size achieves an 80% probability of detecting a 6% difference between women and men in the prevalence of adequate glucose control (two-sided significance level).

Comparisons were stratified by sex. Continuous variables were presented as means with standard deviations. Obesity was included as a categorical variable following the East Asian ethnicity–specific definition (BMI > 25 kg/m^2^). Baseline characteristics were compared using the chi-square or Fisher’s exact tests for categorical variables and Student’s *t*-test or Wilcoxon rank sum test for continuous variables. The relationships between clinical variables and achievement of an HbA1c level below 6.5% after 1 year were analyzed. To account for the nested random effect of primary clinic, generalized linear mixed models (GLMMs) were used. Three models were applied to identify the effects of multiple covariates: Model 0 included the random effect of primary clinic. Model 1 added baseline characteristics to Model 0, including age group, obesity, current smoking, and alcohol use status, initial HbA1c level, diabetes symptoms, Medical Aid use status, underlying medical conditions and aspirin use status. Model 2 added the initially prescribed OHA to Model 1. Adjusted odds ratios (ORs) for achieving the target HbA1c level after 1 year were compared between women and men. All analyses were performed using SAS software (ver. 9.4; SAS Institute Inc, Cary, NC).

## Results

Among the 42 physicians initially recruited, 36 (86%) sent 2,264 CRFs to the research team. Eleven cases (four men and seven women, P = 0.220) were excluded, nine because they did not receive any oral hypoglycemic medication during the first year of diabetes management and two because they were younger than 18 years. Ultimately, 2,253 diabetes patients (949 women and 1,304 men) were included in the analysis.

Table 1 shows the patients’ baseline demographics and clinical presentations by sex. When baseline characteristics were compared by gender, women had an older average age at the time of diagnosis and were more likely to have polyuria (25.5% of women vs. 20.7% of men). Men were more likely to be obese (45.4% of men vs. 38.2% of women), current smokers (73.0% of men vs. 27.4% of women), heavy alcohol drinkers (12.0% of men vs. 1.1% of women), and to complain of weight loss (17.8% of men vs. 13.2% of women) or neuropathy (1.8% of men vs. 0.8% of women), and had higher initial HbA1c and fasting blood glucose levels. There were no significant differences by gender in average BMI, use of Medical Aid, asymptomatic cases and preexisting medical conditions (hypertension, dyslipidemia, coronary heart disease and aspirin intake). Metformin monotherapy was administered initially in around half of the cases, and 46.5% of the single-pill combination OHA regimens were accounted for by metformin+sulfonylurea. There were no differences in initial OHA prescription between men and women after adjusting for baseline characteristics.

**Table 1:** Comparison of baseline demographics and clinical presentations between women and men diagnosed with type-2 diabetes at primary care clinics (N = 2,253).

Variables	Women (n = 949)	Men (n = 1,304)	P value^a^
Age at time of diagnosis (years)	57.3 ± 11.0	53.3 ± 10.9	< .001
BMI (kg/m^2^)	25.3 ± 3.3	25.3 ± 2.9	0.676
Obesity (%)	38.2 (365)	45.6 (594)	0.001
Current smoking (%)	27.4 (260)	73.0 (952)	< .001
Current alcohol use	1.1 (10)	12.0 (157)	< .001
Medical Aid (%)	6.1 (52)	4.1 (53)	0.231
*Clinical presentation*			
Asymptomatic	45.7 (436)	45.6 (595)	0.938
Symptomatic (multiple)			
Polydipsia (%)	21.8 (208)	18.9 (246)	0.081
Polyuria (%)	25.5 (243)	20.7 (270)	0.007
Polyphagia (%)	20.8 (199)	19.6 (255)	0.431
Weight loss (%)	13.2 (126)	17.8 (232)	0.003
Visual impairment (%)	1.5 (14)	1.4 (18)	0.858
Neuropathy (%)	0.8 (8)	1.8 (24)	0.047
Other (%)	3.0 (29)	3.9 (51)	0.273
Unknown (%)	15.1 (144)	13.9 (181)	0.753
*Medical history*			
Hypertension	49.5 (470)	48.5 (632)	0.595
Dyslipidemia	45.4 (431)	43.4 (566)	0.327
Coronary heart disease	2.7 (26)	2.6 (34)	0.843
Aspirin	19.9 (189)	23.4 (305)	0.051
*Laboratory finding*			
Fasting blood glucose (mg/dL)	172.5 ± 61.4	183.8 ± 71.6	< .001
HbA1c (%)	8.2 ± 1.6	8.3 ± 1.7	0.041

BMI, body mass index; HbA1c, hemoglobin A1c

^**a**^ P values are for differences.

^b^ Age, fasting blood glucose and HbA1c are presented as mean ± standard deviation. Other variables are presented as percent (number).

Table 2 summarizes the management scheme for type-2 diabetes patients in the participating primary clinics. About one third of the patients were screened for diabetes complications at their initial visit (36.4% of the total population). Metformin was prescribed in the form of monotherapy or single-pill combinations to most patients. There were no significant differences between men and women in the rate of screening for diabetes complications at the initial visit, prescribed OHAs, total number of visits or HbA1c level at the end of the first year of management.

**Table 2:** Comparison of diabetes management between women and men diagnosed with type-2 diabetes at primary care clinics (N = 2,253).

	Women (n = 949)	Men (n = 1,304)	P value^b^
Screened for diabetes complications	35.9 (341)	36.8 (480)	0.702
*Prescribed oral hypoglycemic agent*			
Metformin	48.1 (456)	45.6 (594)	0.566
Sulfonylurea	23.3 (221)	25.5 (333)	
Other monotherapy	3.3 (31)	3.2 (42)	
Single-pill combination 1 (Metformin + Sulfonylurea)	11.0 (104)	12.6 (164)	
Single-pill combination 2 (Metformin + DPP-IV^a^ inhibitor)	6.0 (57)	5.2 (68)	
Other single-pill combination	8.4 (80)	7.9 (103)	
Number of visits^c^	12.2 (median: 10)	12.1 (median:10)	0.178

Variables are presented as percent (number) except when otherwise specified.

^a^ DPP-IV, dipeptidyl peptidase-4.

^b^ P value for chi-square tests

^c^ Number of visits during 1 year of diabetes management. P value for Wilcoxon rank-rum test

**Fig 1:**
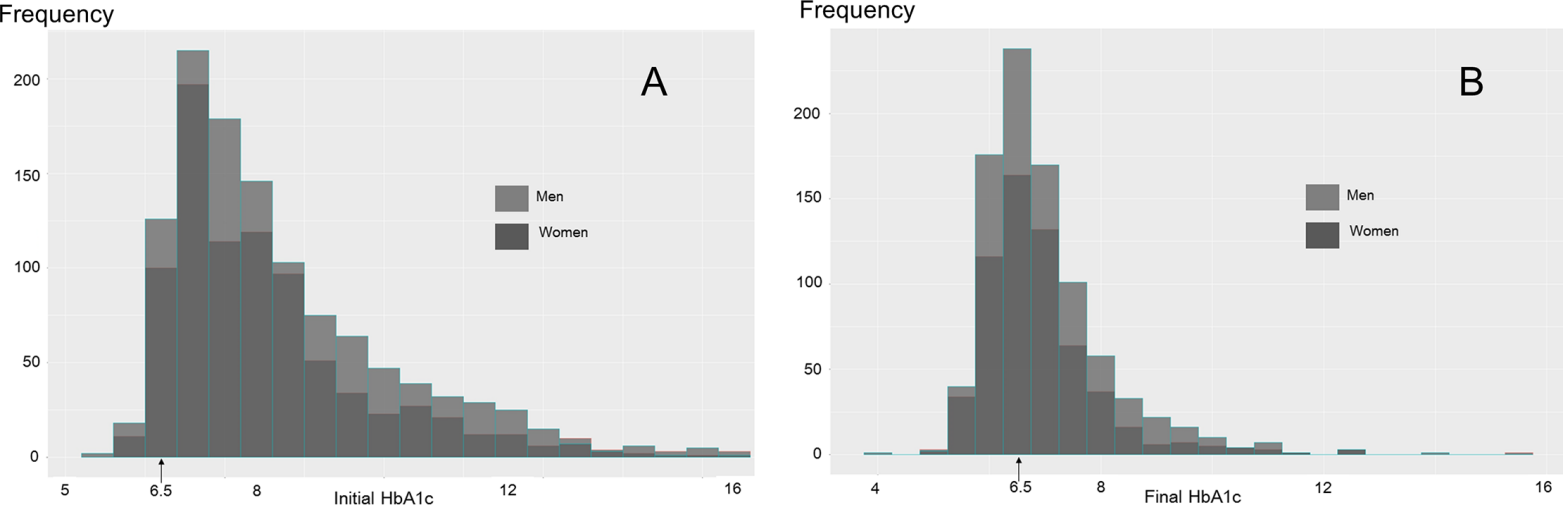
Hemoglobin A1c (HbA1c) levels of women and men diagnosed with type-2 diabetes at primary care clinics (N = 2,253); (A) Initial HbA1c; and (B) Final HbA1c after 1 year.

The initial and final (after 1 year of diabetes management) HbA1c levels were similar between women and men (Fig 1).

Within the study population, 38.9% (369/949) of women and 40.6% (529/1,304) of men achieved the target HbA1c after 1 year of diabetes management. Table 3 compares the adjusted ORs for achieving the target HbA1c level of women and men. In the model, which considers the fixed effect of gender and random effect of primary care clinic, the OR for achieving the target HbA1c level after 1 year of diabetes management was not significantly different between women and men (Model 0). However, after controlling for baseline covariates, women showed lower ORs for HbA1c target achievement than men (Model 1). Lower ORs for women were also observed in the model including prescribed OHA (Model 2). The random effect of primary clinic was significant in all three models.

**Table 3:** Odds ratios for achieving the target HbA1c after 1 year of diabetes management (N = 2,253).

	Model 0 OR [95% CI]	Model 1 OR [95% CI]	Model 2 OR [95% CI]
Women	0.85 [0.70, 1.03]	0.72 [0.56, 0.92]	0.70 [0.55, 0.90]
*Age group*			
< 45 years	-	1.00	1.00 (reference)
46–55 years	-	1.00 [0.72, 1.39]	0.96 [0.64, 1.46]
56–65 years	-	0.92 [0.65, 1.30]	0.79 [0.52, 1.21]
66–75 years	-	0.87 [0.58, 1.30]	0.79 [0.49, 1.27]
> 75 years	-	0.73 [0.40, 1.35]	0.86 [0.43, 1.70]
Obesity (BMI > 25kg/m^2^)	-	0.88 [0.68, 1.15]	0.87 [0.65, 1.17]
Current smoking	-	1.28 [1.00, 1.65]	1.32 [1.00, 1.74]
Current alcohol drinking	-	0.88 [0.55, 1.39]	0.55 [0.19, 1.56]
Initial HbA1c	-	0.75 [0.69, 0.81]	0.71 [0.64, 0.79]
Asymptomatic	-	0.92 [0.70, 1.21]	0.91 [0.68, 1.23]
Medical Aid	-	1.12 [0.58, 2.17]	0.99 [0.48, 2.03]
Hypertension	-	1.06 [0.83, 1.37]	1.08 [0.82, 1.43]
Dyslipidemia	-	0.93 [0.74, 1.17]	0.96 [0.75, 1.24]
Coronary disorder	-	0.36 [0.18, 0.74]	0.23 [0.10, 0.53]
Aspirin	-	0.91 [0.69, 1.22]	1.16 [0.83, 1.61]
*Oral hypoglycemic agents*			
Metformin	-	-	1.00 (reference)
Sulfonylurea	-	-	0.78 [0.56, 1.10]
Other monotherapy	-	-	0.82 [0.38, 1.73]
Single-pill combination 1 (Metformin + Sulfonylurea)	-	-	1.01 [0.67, 1.50]
Single-pill combination 2 (Metformin + DPP-IV inhibitor)	-	-	0.88 [0.53, 1.46]
Other single-pill combination	-	-	1.48 [0.96, 2.28]
*Random effect of primary clinic*			
Variance (Standard deviation)	1.53 (1.23)	1.46 (1.21)	1.56 (1.25)
AIC	1927.3	1965.0	1957.2

OR, odds ratio; CI, confidence interval; DPP-IV, dipeptidyl peptidase-4; AIC, Akaike information criterion. ORs are adjusted values.

Model 0 included the random effect of primary care center. Model 1 added age group, obesity, current smoking, alcohol drinking, initial HbA1c, diabetes symptoms, Medical Aid use, hypertension, dyslipidemia, coronary disorder, and aspirin use status to Model 0. Model 2 added prescribed OHA to Model 1.

## Discussion

This study revealed that among type-2 diabetic patients newly diagnosed at primary clinics, women are less likely than men to reach glycemic control targets after 1 year, without significant differences in diabetes management including hypoglycemic therapies. This finding suggests possible sex differences in glycemic control and the need for a sex-specific approach. As less optimal glycemic control in women has not been clearly addressed in most studies of diabetes management, this finding could be critical for the management of women with type-2 diabetes.

The glycemic target achievement rate was about 39% in this study, which is higher than those in other studies. Previous studies reported that 21.4–30% of patients achieved a target of HbA1c 6.5% or less after 48–52 weeks of OHA monotherapy [19, 20]. Several differences in the diabetes care regimen might have led to the better outcome achieved in our population. Metformin and sulfonylurea were the most commonly prescribed monotherapies in this study, which is consistent with recent Korean national data [21]. In Korea, diabetic patients generally make more frequent (average of 12.2 per year) outpatient visits to health institutions versus patients in previous studies done in other countries [22–24]. This is probably due to Korea’s universal diabetes management program, which subsidizes primary care outpatient service costs [25]. Furthermore, as the study design is retrospective, cases with good medication compliance were more likely to be included, and these with bad medication compliance were more likely to be excluded, than in equivalent prospective clinical trials.

Previous studies have reported differences in the prevalence of several cardiovascular risk factors, such as hyperglycemia, dyslipidemia and obesity, according to sex [26]. Among diabetic patients, overweight or obesity are more prevalent in women than in men [27, 28]. A meta-analysis of 37 prospective cohort studies showed that women with diabetes had higher blood pressure and lipid levels than men with diabetes; similarly, greater differences between women with and without diabetes than in men with and without diabetes were observed [6]. A study of European dyslipidemic patients showed higher total cholesterol and higher high-density lipoprotein cholesterol (HDL-C) levels, and a lower triglyceride level, in women than in men [29]. Sex differences in the clinical presentation of diabetes may be mediated by differences in metabolic profiles, such as the presence of dyslipidemia; the differences in lipid profile were not evaluated in this study. Considering that dyslipidemia is related to poor glycemic control [30], future studies on suboptimal glycemic control among women should include lipid analyses.

To date, studies on sex differences in the management of type-2 diabetes have shown inconsistent results. In a study by Fitzgerald et al., attitudes and adherence to self-care regimens were not significantly different between male and female type-2 diabetes patients [31]. A study of Medicare patients in the United States showed that women were more likely to receive HbA1c screening or eye examinations than men [12]. Rossi et al. reported significant sex differences in the treatment of risk factors for cardiovascular complications among diabetes patients [32]. Another report showed that women with type-2 diabetes were less likely to be monitored regarding their cholesterol or blood pressure, and to reach the cholesterol target, compared to men [33, 34]. In our study population, the frequency rates of initial screening for complications and outpatient monitoring were not different between women and men. In the statistical model, a positive relationship was observed between achieving the HbA1c target and current smoking. This may indicate that men had better results than women in our study due to the effects of smoking cessation, although data on smoking cessation after 1 year were not available. Because of different population characteristics, sex differences in diabetes management would vary depending on the study setting.

The possibility of sex differences in diabetes management is important because women and men with diabetes have been found to have different disease outcomes [35]. Women with diabetes are more likely to experience side effects from OHAs, as well as dyslipidemia and hypoglycemic events [18, 36]. A large cohort study in Italy found that among type-2 diabetes patients, women were less likely than men to reach low density lipoprotein-cholesterol targets [34, 37]. In a study of patients with acute coronary syndromes, diabetes was associated with a higher risk of fatal coronary heart disease or in-hospital mortality in women than in men [6, 38]. Although it is not clear whether these differences are biological or socio-behavioral in origin, the mechanisms underlying these sex-based differences have several determinants (e.g., drug compliance, lifestyle modifications, doctor–patient relationship, etc.) that were not included in this study and thus should be explored further.

Due to the retrospective cohort design used for this study, there were inherent biases in the selection of the study population. Because the patients who were lost to follow-up were not investigated, the findings are not generalizable to the general population. Furthermore, the management and treatment goals of the participating primary care physicians were not standardized. However, since the KDA have recommended an HbA1c level below 6.5% for type-2 diabetes since 2007, they would have had this glycemic control target in common before the study. In addition, medication regimens may have been changed during follow-up, which might have affected treatment success. Furthermore, the OHA doses were not measured. These factors may have mediated the differences in glucose control observed between the sexes, and should be explored in a future study. However, by including more than 2,000 patients with newly diagnosed with type-2 diabetes, the results of this study are still important for primary practitioners treating diabetic patients.

## Conclusions

In conclusion, among newly diagnosed type-2 diabetic patients in participating primary clinics, female patients were less likely to achieve the target HbA1c level after 1 year of diabetes management. For more effective diabetic management, a sex-specific management protocol that considers the differences in clinical features between women and men is needed.

## Supporting information

S1 File Dataset used in the study. (CSV)
